# Significant weight loss in breastfed term infants readmitted for hyperbilirubinemia

**DOI:** 10.1186/1471-2431-9-82

**Published:** 2009-12-31

**Authors:** Ariel A Salas, Jorge Salazar, Claudia V Burgoa, Carlos A De-Villegas, Valeria Quevedo, Amed Soliz

**Affiliations:** 1 Division of Pediatrics, Caja Petrolera de Salud Clinic, 2525 Arce Ave, San Jorge, La Paz, Bolivia; 2 Division of Neonatology, Miami Children's Hospital, Miami, FL, USA

## Abstract

**Background:**

Weight loss of greater than 7% from birth weight indicates possible feeding problems. Inadequate oral intake causes weight loss and increases the bilirubin enterohepatic circulation. The objective of this study was to describe the association between total serum bilirubin (TSB) levels and weight loss in healthy term infants readmitted for hyperbilirubinemia after birth hospitalization.

**Methods:**

We reviewed medical records of breastfed term infants who received phototherapy according to TSB levels readmitted to Caja Petrolera de Salud Clinic in La Paz, Bolivia during January 2005 through October 2008.

**Results:**

Seventy-nine infants were studied (64.6% were males). The hyperbilirubinemia readmission rate was 5% among breastfed infants. Term infants were readmitted at a median age of 4 days. Mean TSB level was 18.6 ± 3 mg/dL. Thirty (38%) had significant weight loss. A weak correlation between TSB levels and percent of weight loss was identified (r = 0.20; p < 0.05). The frequency of severe hyperbilirubinemia (> 20 mg/dL) was notably higher among infants with significant weight loss (46.7% vs. 18.4%; p < 0.05). The risk of having severe hyperbilirubinemia was approximately 4 times greater for infants with significant weight loss (OR: 3.9; 95% CI: 1.4-10.8; p < 0.05).

**Conclusions:**

Significant weight loss could be a useful parameter to identify breastfed term infants at risk of severe hyperbilirubinemia either during birth hospitalization or outpatient follow-up visits in settings where routine pre-discharge TSB levels have not been implemented yet.

## Background

In the current worldwide context of short postpartum hospital stays, it is important to assess factors associated with potentially preventable causes of newborn readmissions [1-6]. Hyperbilirubinemia and feeding difficulties with or without dehydration are the most frequent indications for readmission in the first 2 weeks of life [6,7] and strongly related each other due to inadequate oral intake, particularly in term infants [8-11]. Exclusively breastfed healthy term infants in whom breastfeeding has not been well established by the time of discharge are at greater risk of poor caloric intake, dehydration associated with decreased volume and frequency, and the secondary delayed gastrointestinal motility determines an increase in the enterohepatic circulation of bilirubin [8,12,13]. Weight loss in the infant of greater than 7% from birth weight indicates possible breastfeeding problems [14]. Several studies have previously reported significant weight loss in patients with extreme hyperbilirubinemia [15,16]; however, only few have analyzed separately this association in breastfed otherwise healthy term infants. The objective of this study was to determine the overall readmission rate due to hyperbilirubinemia and to describe the association between total serum bilirubin (TSB) levels and weight loss during the first two weeks of life in breastfed term infants who were discharged home after birth hospitalization and considered to be well infants.

## Methods

This retrospective study included breastfed otherwise healthy term infants readmitted for hyperbilirubinemia during their first two weeks of life after birth hospitalization at Caja Petrolera de Salud Clinic, a tertiary care facility in La Paz, Bolivia from January 2005 through October 2008. In our nursery, neonates are routinely discharged around 48 hours after vaginal delivery, and those of mothers who had undergone cesarean section at 72-96 hours. Breastfeeding is encouraged. Before discharge, all newborns are evaluated for clinical jaundice through skin color observation. Outpatient follow-up visits are scheduled based on physician's criteria in the absence of systematic follow-up protocols in the unit. Pre-discharge bilirubin screening has not been implemented yet.

A readmission was defined as admission of an infant for hyperbilirubinemia after a first hospital discharge diagnosis of healthy term infant. Readmission rate was calculated by using the number of readmissions as the numerator, divided by the total number of healthy term infants born during the period of study. To avoid potential confounding causes of hyperbilirubinemia readmission, we excluded infants with hemolytic disease, infection, major congenital anomalies, respiratory distress, and feeding intolerance. Cephalohematoma and mild bruising were also exclusion criteria.

On readmission, all patients had criteria for phototherapy according to total serum bilirubin (TSB) levels [12]. TSB was measured by colorimetric methods using centrifuged venous blood samples. Only breastfed term (39-41 weeks) infants with a birth weight > 2500 g were included in this study. No tests for 6GPD deficiency were performed in these patients. Birth weight was obtained using an electronic baby weighing scale with a precision of 5 g (Seca 728 Ultimate Digital Baby Scale, Seca Corporation, Hamburg, Germany). The same instrument was used to determine the weight on readmission. Significant weight loss was defined as weight loss from birth weight greater than 7%. A bilirubin level ≥ 20 mg/dL (342 μmol/L) was chosen to define severe hyperbilirubinemia since an infant with this degree of jaundice is thought to be at major risk for neurologic damage [9,17-19].

The chart review was performed with approval of Caja Petrolera de Salud Research & Ethics Committee. Statistical comparisons were done using *t*-tests for continuous variables and *χ*^2 ^tests for categorical variables. Pearson correlation was calculated to compare TSB levels and percent of weight loss from birth. The risk was estimated by odds ratio (OR) and confidence intervals (CI) using contingency tables All statistical tests were two-tailed and P-values < 0.05 were considered statistically significant. The data were analyzed using SPSS 17.0 for Windows.

## Results

From a population of 2140 live term births, 137 (6.4%) term infants were readmitted for hyperbilirubinemia during their first two weeks of life. About 90% of these neonates were exclusively breastfed infants. Fourteen infants were excluded (6 patients had simultaneously diagnosis of infection, 4 had minor congenital anomalies, 3 had hemolytic disease, and one had a cephalohematoma). One hundred eight infants met the criteria of being exclusively breastfed otherwise healthy term infants. Of these infants, only 79 infants had sufficient data on medical records for analysis. Table 1 summarizes clinical and demographic characteristics of these infants according to the severity of hyperbilirubinemia on admission.

**Table 1:** Characteristics of exclusively breastfed term infants readmitted for hyperbilirubinemia (n = 79)

	Severe hyperbilirubinemia (≥ 20 mg/dL) n = 23	Significant hyperbilirubinemia (< 20 mg/dL) n = 56	p
**Perinatal history**			

Birth weight (g) Mean ± SD	3060 ± 482	3197 ± 505	0.69

Gender male/female (%)	69.6/30.4	62.5/37.5	0.55

Maternal age (yr) Mean ± SD	29.4 ± 5.6	30.3 ± 6.1	0.50

Birth from vaginal delivery (%)	39.1	37.5	0.89

**On admission**			

Age (d) Mean ± SD	6.3 ± 3.6	4.0 ± 2.1	< 0.05

Weight (g) Mean ± SD	2920 ± 367	2880 ± 475	0.69

Infant's weight loss from birth (g) Mean ± SD	277 ± 221	179 ± 149	< 0.05

Percent of weight loss from birth Mean ± SD	8.8 ± 4.8	5.9 ± 4.7	< 0.05

Length of hospital stay (d)	3.0 ± 1.6	2.4 ± 1.1	0.13

Significant weight loss (%)	60.9	28.6	< 0.05

**Figure 1:**
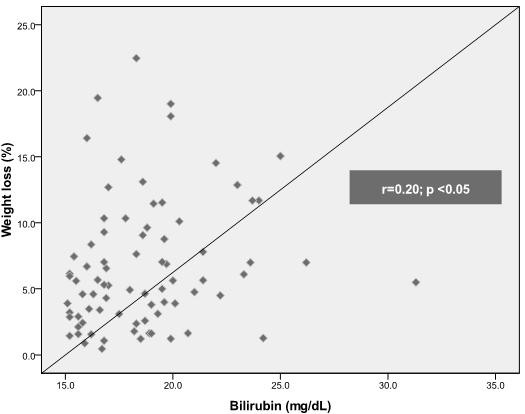
**Linear correlation between TSB levels and percent of weight loss in exclusively breastfed term infants readmitted for hyperbilirubinemia**.

Breastfed term infants were readmitted for hyperbilirubinemia at a median age of 4.7 days. Approximately two-thirds of these infants were males (64.6%). Mean TSB level on admission was 18.6 ± 3.0 mg/dL (range: 15.1 - 31.3 mg/dL). Thirty (38%) infants readmitted for hyperbilirubinemia had significant weight loss. Two thirds of these patients (60%) had weight loss >10%. Mean TSB levels in infants with hyperbilirubinemia alone was significantly lower than mean TSB levels in infants with hyperbilirubinemia and significant weight loss (18.0 vs. 19.5 mg/dl; p < 0.05). Figure 1 shows a weak positive correlation between TSB levels and percent of weight loss (r = 0.20; p < 0.05). The frequency of severe hyperbilirubinemia was higher among infants with significant weight loss (46.7% vs. 18.4%; p < 0.05). The risk of having severe hyperbilirubinemia was approximately 4 times greater for infants with significant weight loss compared with infants who had acceptable weight loss (OR: 3.9; 95% CI: 1.4-10.8; p < 0.05). The risk was greater for infants who had weight loss > 10% (OR: 4.2; 95% CI: 1.4-12.7; p < 0.05). The length of hospital stay (median: 2 days) did not differ between groups (3.0 vs. 2.4 days; p = 0.13). The route of delivery did not influence significantly on differences between TSB levels (p = 0.65), age at admission (p = 0.93), and percent of weight loss at admission (p = 0.66). Infants with severe hyperbilirubinemia were readmitted at a mean of 6.3 days and those with hyperbilirubinemia alone at 4 days (p < 0.05). Extreme hyperbilirubinemia (>25 mg/dL) was identified in three patients (3.8%).

## Discussion

In this study, significant weight loss was notably associated with hyperbilirubinemia readmission in exclusively breastfed otherwise healthy term infants. The overall readmission rate was 64 per 1000 term infants, and approximately 50 per 1000 exclusively breastfed term infants. Hyperbilirubinemia readmission rates in term infants usually vary from 2 to 21.7 per 1000 [2-6,10,20,21]. Exclusive breastfeeding is not only a major risk factor for hyperbilirubinemia but also for dehydration, particularly if nursing is not going well and weight loss is excessive [12,22-26]. Weight loss > 5% was observed in about 25% of breastfed infants during their first 24 hours of life [27]. Approximately one-third of breastfed term infants readmitted for hyperbilirubinemia (mean TSB level of 22.8 mg/dL) showed to have weight loss from birth > 12% [6]. In addition, breastfed infants with significant hyperbilirubinemia (> 12.9 mg/dL) showed greater weight loss from birth than bottle-fed infants (6.9% vs. 4.2%) [11]. Also, significant hyperbilirubinemia was associated with a greater weight loss after 72 hours of life (8.0% vs. 6.4%) [8]. Fasting and poor caloric intakes seem to have a greater effect on the regulation of serum bilirubin than breastfeeding *per se *[8,11]. We found a significant difference in percent of weight loss between infants with severe and significant hyperbilirubinemia (8.8% vs. 5.9%, respectively). Our study also showed that approximately 60% of infants readmitted for severe hyperbilirubinemia had significant weight loss.

A mean age at admission of 4.7 days found in our study is comparable to previous reports [1,4-6,10,23,28]. Breastfed infants experience their maximum weight loss by day 3 [12]. In a previous study, the majority of newborns readmitted for feeding problems were 4 to 7 days old, and many had concurrent dehydration and jaundice (34.3%) [26]. Since infants with severe hyperbilirubinemia were readmitted approximately 2 days later than infants with hyperbilirubinemia alone, we could assume that a significant proportion of these infants would have been detected early in follow-up visits based on weight loss from birth according to the weak positive correlation found in this study. This analysis is consistent with the findings of a recent study which demonstrated that if weight loss >10% leads to interventions to improve nutrition and hydration, no association with extreme hyperbilirubinemia is found [13].

Interestingly, the percentage of infants delivered by cesarean section (CS) was unexpectedly higher in this report. Some common indications for CS such as gestational diabetes, pregnancy-induced hypertension, and premature rupture of membranes are risk factors for readmissions in term infants [1,10]. In addition, length of stay after CS is a significant predictor of readmission for breastfeeding difficulties, mainly because lactogenesis was shown to occur later for women after cesarean delivery either for physiologic reasons or because of a delay in the initial feeding after surgery [1]. In contrast, several studies have showed that CS is a protective factor for neonatal hyperbilirubinemia. CS would reduce the risk of admission for increased maternal rest, teaching, and enhanced lactation during the third hospital day [6]. Furthermore, longer inpatient hospital stays have been associated with breastfeeding success after cesarean delivery [29]. Also, it has been suggested that infants born by emergency cesarean section are stressed before birth and, therefore, induce conjugating enzymes before delivery. Finally, less placental transfusion in infants born by cesarean section has also been proposed as a protective factor [8]. In our study, although the history of a cesarean delivery was frequent among term infants readmitted for hyperbilirubinemia, this variable does not seem to affect the severity of weight loss, age of presentation, or TSB levels on admission.

Initially, length of hospital stay (LOS) < 48 hours showed to be a risk factor for hospital readmission due to hyperbilirubinemia [3,4]; however, inadequate nursing seemed to have the greatest impact on hospital readmission since jaundiced infants showed greater weight loss than non-jaundice infants (6.8 vs. 4.0%) [4]. Accordingly, recent regression analysis revealed no increased odds of readmission with LOS < 2 days. Infants delivered vaginally with 1 night of hospital stay and adequate prenatal and postnatal care outside the hospital had no increased risk of readmission [2]. In addition, no significant increased risk of readmission for hyperbilirubinemia was found among infants who were born vaginally and discharged < 24 h after birth[10]. Similarly, no association between dehydration and neonatal or maternal LOS was reported [22]. Finally, early discharge following an uncomplicated postpartum hospital stay appeared to have no independent effect on the risk of readmission in infants with feeding-related problems [26]. Based on these reports, the best intervention would be to help mothers to nurse their infants more effectively from the moment of birth [1]. Outpatient follow-up strategies occurring between 24 and 48 hours after discharge would also prevent dehydration and hyperbilirubinemia [22]. Therefore, discontinuing early hospital discharge practices may not be the best means to decrease the risk of hospital readmission for hyperbilirubinemia [3]. The specific age at time of discharge after their birth hospitalization was not determined in this study, but we agree that improving our follow-up programs will have a greater impact on reducing the risk of severe hyperbilirubinemia rather than modifying the current global tendency of shorter newborn hospital stays.

A major limitation of this analysis is the lack of information regarding infant feeding. The second major limitation of this report is the absence of data related to the timing and extent of newborn follow-up in the outpatient setting. As mentioned above, the timing of follow-up may reduce readmissions if jaundice and/or feeding problems are caught early enough. One additional limitation is our inability to compare these results with a normal newborn population. Another important consideration is that the use of neonatal readmission as a primary outcome has advantages and disadvantages, which have been extensively reviewed and discussed [1]. Finally, our study was conducted in a high-altitude city (3600 m above sea level). At similar altitude, incidence of neonatal hyperbilirubinemia, defined as TSB levels >12 mg/dl, showed to be approximately four times the incidence reported in the literature for sea level [30].

Despite the limitations of this study, we found that significant weight loss increase approximately 4 times the risk to develop severe non-hemolytic hyperbilirubinemia in breastfed term infants and it seems to be worst when the cut point to define significant weight loss is higher (infants with a weight loss of 10% have odds 4.2 times higher). Both hyperbilirubinemia and feeding problems persist worldwide despite well-intentioned guidelines for care showing that practice related to newborn care and follow-up seem to be resistant to change, particularly in less-developed countries. Our findings also highlight the need for better data about the content of outpatient follow-up visits.

## Conclusions

Significant weight loss reflects feeding problems and seems to be an important factor associated with severe hyperbilirubinemia in breastfed term infants. If these findings are confirmed by large prospective studies in order to determine not only association but also a cause-effect relationship, weight loss from birth could become a useful clinical parameter to identify breastfed term infants at risk of severe hyperbilirubinemia either during birth hospitalization or follow-up visits, particularly in settings where routine pre-discharge TSB levels have not been implemented yet.

## Competing interests

The authors declare that they have no competing interests.

## Authors' contributions

AAS conceived and designed the study and participated in the analysis and writing of the manuscript. JS coordinated the study and participated in the design. CB participated in the statistical analysis. CD participated in collection and interpretation of data. VQ participated in collection and interpretation of data. AS reviewed the manuscript critically for important intellectual content. All authors read and approved the final manuscript.

## Pre-publication history

The pre-publication history for this paper can be accessed here:

http://www.biomedcentral.com/1471-2431/9/82/prepub
